# Rhinovirus Infection in Children with Acute Bronchiolitis and Its Impact on Recurrent Wheezing and Asthma Development

**DOI:** 10.3390/microorganisms8101620

**Published:** 2020-10-21

**Authors:** Carlotta Biagi, Alessandro Rocca, Giulia Poletti, Marianna Fabi, Marcello Lanari

**Affiliations:** Department of Medical and Surgical Sciences, Pediatric Emergency Unit, St Orsola Polyclinic, University of Bologna, 40138 Bologna, Italy; alessandro.rocca4@unibo.it (A.R.); giuliapoletti89@gmail.com (G.P.); marianna.fabi@aosp.bo.it (M.F.); marcello.lanari@unibo.it (M.L.)

**Keywords:** rhinovirus, bronchiolitis, clinical severity, asthma, wheezing, children

## Abstract

Acute bronchiolitis represents the leading cause of hospitalization in infants. Together with a respiratory syncytial virus, rhinovirus (RV) is one of the most common pathogens associated with bronchiolitis, and its genetic diversity (>150 types) makes the recurrence of RV infections each year quite typical. The frequency of RV infection and co-infection with other viruses and its impact on the clinical course of bronchiolitis have been studied by several authors with controversial results. Some studies demonstrate that multiple virus infections result in more severe clinical presentation and a higher risk of complications, whereas other studies suggest no influence on clinical course. Moreover, RV bronchiolitis has been reported to potentially contribute to the development of long-term sequelae, such as recurrent wheezing and asthma, in the pediatric population. In the present review, we summarize the most recent findings of the role of RV infection in children with acute bronchiolitis, its impact on subsequent asthma development, and the implication in clinical practice.

## 1. Introduction

Rhinovirus (RV) is a non-enveloped single-stranded RNA virus belonging to the Enterovirus genus in the Picornaviridae family. It is a highly contagious and ubiquitous virus. Its transmission generally occurs through direct exposure to respiratory droplets/micro-droplets, even though it can also take place via contaminated surfaces, including direct person-to-person contact [1]. In temperate climates (i.e., many areas of the USA and Europe), RV is responsible for annual outbreaks in the period from early fall to the end of spring [2].RV has three different subgroups—A, B, and C—which consist of 80, 30, and 56 types, respectively [3]. The genetic diversity of RV (>150 types) makes the recurrence of RV infections each year quite typical and the development of an effective vaccine very difficult [4]. RV represents the main responsible agent of “common colds”, mostly characterized by rhinorrhea, sore throat, cough, and diffuse malaise. RV can also cause many other upper and lower respiratory tract infections, such as otitis media, croup, pneumonia, and acute bronchiolitis [5,6,7].

Acute bronchiolitis is the most frequent lower respiratory tract infection in children, especially in preterm infants, and represents the leading cause of hospitalization in infants, accounting for 18% of all pediatric hospital admissions in the United States [7,8]. Various definitions of bronchiolitis have been proposed [7,9,10]. Bronchiolitis is defined by the American Academy of Pediatrics (AAP) as a constellation of signs and symptoms, including a viral upper respiratory tract prodrome, followed by increased respiratory effort and wheezing in children under the age of two [9].In Europe, by contrast, the term bronchiolitis is generally referred to as a first episode of acute lower airway infection in infants younger than one year [7]. Clinically, it is characterized by few days of rhinorrhea, fever, and cough, which precede the signs of lower respiratory distress associated with wheeze and/or crackles on chest auscultation. Most children with bronchiolitis have an uneventful course. Hospitalization is required in about 3% of cases, and admission to a Pediatric Intensive Care Unit (PICU) in approximately 2–6% of the hospitalized cases [11]. According to the National Institute for Health and Clinical Excellence (NICE) guidelines, indicators for hospital admission are respiratory rate over 60 breaths/minute, marked chest wall retractions, apnea, an oxygen saturation (SpO2) lower than 92%, central cyanosis, poor oral fluid intake, inability or indifference to eating due to breathlessness [10]. Even if the diagnosis of bronchiolitis is mainly based on history and clinical findings, and the treatment is primarily supportive, the identification of the causative organism should improve the understanding of the disease and open avenues for precision medicine.

During the last 20 years, the methodologies for virus detection—immunofluorescence assay, but also molecular investigations, such as polymerase chain reaction—have improved, increasing the knowledge of the viral agents responsible for acute bronchiolitis. These techniques have led to the identification of the main responsible pathogen, respiratory syncytial virus (RSV), accounting for 70–80% of bronchiolitis, followed by RV [12]. Approximately 20–40% of children with bronchiolitis seem to be infected by RV [13]. Other viruses, such as adenovirus, influenza, parainfluenza, metapneumovirus, human bocavirus, and human coronavirus, are less frequently implicated. Up to 30% of hospitalized infants with bronchiolitis have multiple respiratory virus co-infections [14]. Some viruses might be detected because of colonization, prolonged viral-shedding post-infection, or incubation before clinical infection. Indeed, respiratory viruses have been found in up to 40% of asymptomatic children. According to this finding, the interpretation of multiple coexisting viruses in symptomatic subjects should be interpreted with caution.

The frequency of RV infection and co-infection with other viruses and its impact on the clinical course of bronchiolitis have been studied by several authors with conflicting evidence. Moreover, RV bronchiolitis has been reported to potentially contribute to the development of long-term sequelae, such as recurrent wheezing and asthma, in childhood. 

The objective of this review is to provide an overview of the role of RV infection in children with acute bronchiolitis and to depict its potential impact on the clinical course of the illness and the subsequent development of asthma in the pediatric population.

## 2. Rhinovirus Infection and Bronchiolitis Severity

Many studies have investigated whether the severity of acute bronchiolitis—mainly measured by clinical score indexes (CSIs), oxygen requirement, ventilatory support, Pediatric Intensive Care Unit (PICU) admission, and length of hospital stay (LOS)—is associated with specific viral infections or co-infections, with controversial results.

The results of the most important studies analyzing the correlation between the pathogen involved (RV vs. other respiratory viruses) and the severity of bronchiolitis are summarized in Table 1.Only the studies published in the last 20 years and enrolling more than 100 children are considered and described below.

### 2.1. RV Infection vs. Other Sole Pathogen Infections

Several studies have investigated the relationship between specific viral pathogens and bronchiolitis CSI based on different symptoms and signs, mainly heart and respiratory rate, clinical signs of respiratory distress, the difficulty in feeding, and oxygen saturation [15,19,20,23,24,34]. As shown in Table 1, the majority of the studies reported that RV is associated with a milder CSI compared to other viral agents [19,20,24]. In the single-center prospective study of Midulla and colleagues, enrolling 182 infants hospitalized with bronchiolitis, RV appeared to be associated with a lower CSI value at hospital admission when compared to other viral agents, firstly RSV (*p* = 0.05) [19]. Similarly, Miller and colleagues assessed the relationship between the viral pathogen and the bronchiolitis severity in 455 hospitalized infants over 4 years (2004–2008). They found that RV was related to a lower CSI when compared to other viral pathogens (*p* < 0.001) [20]. A similar result emerged when the same authors specifically compared RV and RSV bronchiolitis, concluding that RV was once again associated with lower CSI (*p* = 0.013) [24]. On the contrary, the group of Papadopoulos found that RV was associated with the risk of a higher CSI at hospital admission when compared to other viral groups in 118 children with bronchiolitis, both at the univariate analysis (*p* = 0.004) and multivariate analysis (*p* = 0.022) [15]. Corresponding results emerged from another single-center retrospective study analyzing data of 180 infants with bronchiolitis. However, in this study, RV infection was associated with a more severe CSI than the other viral types only at the univariate analysis (*p* = 0.041), while no significant differences emerged at the multivariate logistic regression analysis [23]. The more recent research focusing on this matter was the study retrospectively conducted by Petrarca et al. They enrolled 486 patients during 12 consecutive epidemic seasons without finding significant differences in CSI between RV and RSV-bronchiolitis [34].

The great heterogeneity of the reported results can be justified, at least in part, by the adoption of different CSIs that do not always consider the same parameters and whose point’s range significantly differ from each other. Moreover, many of these scores have not been formally validated, limiting their role in the assessment of the severity of bronchiolitis. Therefore, many authors have considered other more objective criteria for the evaluation of bronchiolitis severity, mainly represented by oxygen therapy, ventilatory support, LOS, and PICU admission.

Regarding oxygen therapy, RV infection seems to be associated with lower oxygen requirement and duration. In a cohort of 455 infants with acute bronchiolitis described by Miller et al., RV infection was associated with a lower frequency of supplemental oxygen requirement than infants with other viral pathogens (*p* < 0.001) [20]. Moreover, Marguet and colleagues found that RVinfection decreased the risk of oxygen requirement compared to RSV-infection (OR 0.29, 95% CI 0.09–0.90 at the logistic regression) in a prospective multicenter study enrolling 209 infants [17]. Nevertheless, no significant differences emerged in the more recent and larger (486 cases), albeit retrospective, research of Petrarca et al. [34]. Similarly, no differences among viral pathogens emerged concerning ventilatory support, intended as continuous positive airway pressure (CPAP) and/or intubation requirement during the hospitalization [22,29,37]. These data resulted from the Multicenter Airway Research Collaboration (MARC), a program of the Emergency Medicine Network, which was responsible for the enrollment of the largest cohorts of hospitalized infants with bronchiolitis, prospectively studied in multiple sites in the USA. In 2012, Mansbach et al. analyzed data obtained from the enrollment of 2207 patients during three winter seasons. They found a viral pathogen in 2068 of cases and compared single-RV to single-RSV infection, as well as co-infections, without discovering significant differences in terms of CPAP/intubation requirement [22]. In more recent years, a similar study was performed, also in Finland. In 2015, Jarttiet al. collected data from both the enrolled American and Finnish cohorts, amounting to 2615 children <2 years of age, of whom 694 documented RV infection. To the best of our knowledge, this study was the only one to investigate the relationship between RV viral load and severity of the disease, and no significant differences emerged in CPAP or intubation requirement [29]. Similarly, Hasegawa et al. compared enrolled American (1016 patients, of which 197 were RV-infected) and Finnish (408 patients, of which 109 were RV-infected) cohorts of infants. Here, they specifically identified also the RV-A, RV-B, and RV-C types responsible for the collected bronchiolitis (47%, 6%, and 47% in the American group and 23%, 3%, and 74% in the Finnish group, respectively). No significant differences in terms of CPAP and/or intubation requirement emerged between the three different RV types in both analyzed cohorts [37]. Finally, also in a prospective multicenter study focusing on 363 hospitalized infants with moderate-severe bronchiolitis, no significant differences appeared between RV infection—even when they considered viral types—and other single virus-infections regarding either oxygen requirement, ventilation need, or nasogastric feeding [31].

Furthermore, many studies have analyzed the hospital LOS as an index of bronchiolitis severity. Only one study has reported a longer LOS in RV bronchiolitis compared to RSV bronchiolitis: Paul and colleagues enrolled a cohort of 319 hospitalized children for acute bronchiolitis, focusing on single RV or single RSV infections (65 cases for the former, 162 cases for the latter). Analyzing the mean LOS as clinical outcome emerged a greater LOS in the RV group compared with the RSV group (*p* = 0.032) [32]. More studies have observed instead a correlation between RV infection and a lower LOS when this virus is compared to other viral pathogens, especially RSV [17,19,26,36]. In the prospective single-center study conducted in 2010 (182 infants), Midulla et al. found that RV infection had a lower mean LOS than RSV-infection (*p* = 0.05) [19]. Jartti and colleagues came to comparable results on a cohort of 408 children comparing RV infections to RSV infections in terms of LOS shorter or longer than three days (*p* = 0.03) [26]. Similarly, Marguet et al. studied 209 infants considering the duration of LOS > 5 days as an outcome, finding that RV infection decreased the risk of prolonged LOS compared to other viral infections, including RSV-bronchiolitis (OR 0.11, 95% CI 0.03–0.37) [17]. Finally, in 2019, Bergroth and colleagues analyzed once again a Finnish cohort obtained from the MARC protocol study, specifically focusing on the three RV types. They demonstrated a correlation between RV-A and RV-C (but not RV-B) infection and a lower LOS when compared to RSV-infection (*p* = 0.003). This result appeared limited by the very low frequency of detected RV-B infection (3%) [36]. On the contrary, many other studies have not found significant differences in terms of LOS between RV bronchiolitis and other viral group-infection [18,20,25,28,30,31,33,34].

Analyzing the rate of admission to PICU in bronchiolitis, Bergroth et al. found that none of the 101 RV-infected patients of their cohort needed intensive care compared to 9% of the 145 RSV-bronchiolitis (*p* = 0.02) [36]. Nevertheless, other studies have reported no significant differences in terms of admission to PICU between the different types of viral infections [27,34,35]. In 2018, Praznik and colleagues conducted a retrospective single-center study analyzing the site management of 761 children with bronchiolitis (138 treated as outpatients, 599 treated in the standard hospital setting, 24 admitted to PICU). Besides, in this study, no differences regarding the causative viruses were found [35]. Moreover, data from the previously cited largest American and Finnish cohorts studies in the context of MARC’s databases—focusing on genomic RV load and RV types—did not find significant differences in terms of PICU admission [29,37].

When taken all into account, these findings seem to support the milder severity course of RV bronchiolitis in comparison with the illness of other viral pathogens, especially RSV. However, it is important to consider the heterogeneity of the reported studies—from the study design (retrospective vs. prospective, case-control. or cross-sectional) to the cohort’s inclusion criteria (such as age, <1 year vs. <2 years)—which may limit the relevance of these results. In particular, the age could represent an important factor, influencing RV and RSV severity. Indeed, RSV dominates in young infants, whereas the prevalence of RV bronchiolitis increases steadily with age [18,19,20,24,26,31,36]. Regarding the studies enrolling children under the age of two, three of them have conducted a parallel analysis using a stricter definition for bronchiolitis (age <12 months) [26,29,36]. No differences are seen in this subset of patients, with the exception of the study of Jartti and colleagues [26]. In this study, RV etiology was associated with shorter hospital LOS compared with RSV in all children with bronchiolitis, but this finding lost its statistical significance in the subset aged <12 months.

### 2.2. Sole RV Infection vs. Multiple Viral Co-Infections

According to the studies reported in Table 1, the frequency of viral co-infections in bronchiolitis varies from 11% to 67% [30,31]. Specifically, the reported frequency of RV + RSV co-infection varies from 2.6% to 62.5% [15,36], while the frequencies of RV + non-RSV viral pathogens co-infections range from 5% to 20% [22,29].

Although it may be intuitive to think that multiple viral bronchiolitis tends to be more severe than single viral illness, only the retrospective study of Richard and colleagues in 2008 found data supporting this hypothesis. In this study, children with viral co-infection (44 patients, 13 of which with RV + RSV bronchiolitis) were associated with a higher risk of admission to PICU at the multivariate analysis (*p* = 0.02). Moreover, the study observed that LOS tended to be prolonged in co-infections, but no statistically significant differences were found [16].

Surprisingly, in a prospective single-center study enrolling 142 hospitalized children with bronchiolitis, co-infections were more frequently associated with mild (no hypoxia or feeding problems) and moderate (oxygen requirement, feeding problems) illness than to severe disease (mechanical ventilation requirement) (*p* = 0.003). In this study, infants with sole RSV infection had the most severe diseases. As suggested by the authors, the young age of these infants might have represented a bias in the results since a young age is a well-known risk factor for severe RSV-bronchiolitis, more important than multiple virus infections [21].

Mansbachet al. described data on 2207 children with bronchiolitis, of which 658 were with multiple infections. They compared RV single infection with RSV single infection, RV + RSV co-infection, RV + non-RSV co-infection, and RSV + non-RV co-infection. This study did not find any significant differences in terms of ventilatory support (intended as CPAP and/or intubation requirement) and admission to PICU but reported the association between RV + RSV co-infection and longer LOS (*p* = 0.04). Regarding this peculiar result, the authors suggested that it could be determined by the specific inflammatory property of RSV, which tends to reduce interferon (IFN)-γ response during infection, possibly allowing an enhancement of RV replication. Another suggested hypothesis is that RSV-infected endothelial cells increase the intercellular adhesion molecule-1 (ICAM-1) expression, the major receptor for RV, setting the stage for a more severe RV infection [22].

However, many other studies that have compared RV bronchiolitis with RSV + RV co-infection or other co-infections have not found any statistically significant difference in terms of CSI [34], respiratory support [31,34], admission to PICU [27,34], and LOS [18,25,30,34].

The great heterogeneity of these results may be due to the differences between the described researches, such as study design, sample size, and considered outcomes, but also may be partially related to the fact that RV is a very ubiquitous virus. Its prevalence among asymptomatic children has been reported up to 40% [38], and its detection in nasal specimens, together with other viruses during acute bronchiolitis, may lead to giving RV a causative role that it possibly does not always have [39].

### 2.3. Possible Influencing Factors: The Viral Load and Different Viral Types

Factors that may play a role in the clinical impact of bronchiolitis are the viral load and the type of RV responsible for the infection.

To the best of our knowledge, only two studies have reported up to now data about the correlation between RV viral load and bronchiolitis severity, without finding any statistically significant result [29,31]. Surely, future studies on this topic could add new information about this relevant matter.

Regarding the different RV types, few studies have focused on their clinical impact, probably because of the recent introduction of molecular detection methods. Infection with RV-C has been associated with more-severe lower respiratory tract illness in the pediatric population compared to RV-A [40,41]; however, this finding has not been consistent across studies [38,42,43]. Regarding acute bronchiolitis, RV-C seems clinically similar to RV-A and RV-B [28,31,37]. In 2014, Selvaggiet al. genotyped 40 RV-infected patients, discovering a frequency of 45% of RV-A subtype and 55% of RV-C type [28]. Skjerven and colleagues in 2016 similarly reported a higher prevalence of RV-C type in their 122 RV-infected patients (71.3% vs. 28.7% of RV-A/B types) [31]. In both studies, no significant differences in terms of hospital LOS and the level of supportive care emerged among the viral types. Similarly, Hasegawa et al. genotyped both the American and Finnish cohorts without discovering significant differences in terms of LOS, PICU admission, and CPAP/intubation requirement between the three different RV types [37]. Bergroth and colleagues genotyped their 101 RV patients and specifically compared them with RSV-bronchiolitis. They found that RV-A and RV-C infections (but not RV-B infection) were associated with lower LOS than RSV-infection (*p* = 0.003) [36]. Nevertheless, the extremely low frequency of detected RV-B type (only three cases) prevents to draw conclusions about the role of this viral species on this matter.

## 3. Long-Term Sequelae of RV Bronchiolitis

Some viral agents, such as RSV, have been associated with the development of asthma in children [44]. In literature, RV bronchiolitis appears more clearly to potentially contribute to the development of recurrent wheezing and asthma in childhood. The results of the most important studies analyzing the correlation between RV infection and preschool wheezing or asthma are summarized in Table 2 and described below. Only researches with clinical outcomes are taken into account.

### 3.1. RV Bronchiolitis and the Risk of Subsequent Wheezing or Asthma

Supposing the role of viral pathogens in asthma development, in 2011, Koponenet al. followed-up a cohort of 205 infants hospitalized for bronchiolitis at <6 months of age. Here, 81% of these patients received a control/telephone interview at 5–6 years of age, focusing on the presence of current asthma during the preceding year [45]. Current asthma was defined in case of a continuous maintenance medication for asthma, doctor-diagnosed wheezing, or prolonged (at least 4 weeks) cough apart from infection. The viral etiology of bronchiolitis was demonstrated in 97% of cases: RSV 70.5%, RV 12.7%, others 16.9%. At the adjusted multivariate analysis, non-RSV bronchiolitis resulted as an independent risk factor for preschool asthma (*p* = 0.01). Although this study did not find a direct link between RV and asthma, the strong correlation between previous non-RSV bronchiolitis and the risk to the development of asthma led to focus the attention on RV, the second viral pathogen responsible for bronchiolitis [45]. In 2014, Midullaet al. published data on a cohort of 313 infants hospitalized for bronchiolitis who underwent yearly follow-up up to three years from the time of discharge. They examined the correlation between viral pathogens (RSV in 73.9% of cases, RV in 14.9% of cases, other viral agents in 21.6% of cases) and the recurrence of wheezing. They observed that RV was the only virus associated with recurrent wheezing (defined as ≥2 episodes in a year for three years) at multivariate analysis (*p* = 0.03). Moreover, they confirmed the lack of association between RSV bronchiolitis and wheezing [46]. On the contrary, the study of Amat and colleagues [47] on a cohort of 154 hospitalized infants for bronchiolitis did not find any difference between the kind of respiratory virus (RSV in 76% of cases, RV in 28.6%, other viruses in 70.1% of cases) and the subsequent development of recurrent bronchial obstruction (intended as ≥3 respiratory symptoms documented in ≥2 times). However, the same study demonstrated that RV + RSV co-infected bronchiolitis was associated with the risk of sensitization to aeroallergens at three years at the multivariate analysis (*p* = 0.02) [47].

Once again, the cohorts enrolled in the context of MARC’s studies have given results also in terms of the correlation between RV bronchiolitis and the development of asthma [36,49]. In 2019, Bergroth and colleagues analyzed the use of asthma control medication four years after hospitalization for bronchiolitis in 349 children. They found that RV infection compared to RSV infection was associated with higher use of asthma control medication in the last year (*p* < 0.001). Moreover, they genotyped RV, finding RV-A type in 24% of RV cases, RV-B in 3% of cases, and RV-C in 73%. At the multivariable analysis, RV-C was specifically associated with a higher risk for the use of asthma control medication four years after severe bronchiolitis (*p* < 0.001), more than RV-A type (*p* = 0.03). The authors supposed that specific RV-C risk genes, such as cadherin-related family members 3, could predispose to the development of asthma after infection with this RV type [36]. In 2020, Mansbach and colleagues analyzed the American MARC’s cohort of 673 hospitalized infants for bronchiolitis, attending two years of follow-up after discharge. Collecting nasopharyngeal aspiration three weeks after the discharge, they focused their attention on the hypothesis that a delayed viral clearance or a sequential infection may be related to the presence of recurrent wheezing by the age of three. Recurrent wheezing was defined as having ≥2 corticosteroid-requiring exacerbations in 6 months or ≥4 wheezing episodes in a year that last ≥1 day and affect sleep. They found that infants with RV bronchiolitis at hospitalization, followed by a new RV infection, had the highest risk of recurrent wheezing (*p* = 0.01). This result was limited by the small number of considered patients (eight infants); however, the authors suggested that it could indicate a specific role by subsequent several RV genotype infections in the genesis of wheezing [49].

Finally, Hunderi and colleagues conducted a prospective multicenter study enrolling 294 hospitalized children with moderate-severe bronchiolitis [48]. Recurrent wheeze was defined as ≥3 episodes of wheezing, including the bronchiolitis at the enrollment. The authors found that recurrent wheezing at the age of two years was not significantly associated with RV or RSV infection, with the RV type or with the viral load during acute bronchiolitis. These results contrasted with previous studies and did not support the hypothesis that RV infection in susceptible infants may predispose to the development of wheezing [48]. However, a longer follow-up period could have provided further insight into the role of RV infection in early sensitization and asthma development.

### 3.2. Potential Role of RV in the Pathogenesis of Asthma

Unlike other respiratory viruses, such as RSV, RV does not have a cytopathic effect and is not able to cause direct airway epithelial cell destruction. However, it alters the epithelial barrier’s function, dissociating the zonula occludens-1 from tight junction complex through the release of reactive oxygen species during viral replication [50]. This alteration can allow the absorption of higher amounts of aeroallergens, strictly related to the development of wheezing and asthma [39]. Moreover, different RV types use several specific vehicles to enter into airway epithelium: RV-A and -B types use ICAM-1 or the low-density lipoprotein receptor (LDL-R), while recently, the cadherin-related family member 3 has been identified as a receptor for RV-C [36,39]. These different ways of access may partially justify the recurrent finding that RV-A and RV-C are more often associated with wheezing in asthma exacerbations [39]. Another peculiar aspect concerns the genetic variations at locus 17q21, which are related to the risk of asthma. Indeed, early-life respiratory wheezing illnesses are a stronger risk factor for asthma in children with 17q21 locus risk variants than in children without it, and this is more evident for episodes triggered by RV than RSV [36,51].

Some immunologic factors of the innate and adaptive immune response result typical of RV infection and may contribute to the development of wheezing or asthma. Recent studies have observed that a Th-2-mediated immune response is more frequent after RV infection. Key factors in this immunologic pattern are interleukin (IL)-4, IL-5, IL-10, IL-13, IL-25, IL-33, and thymic stromal lymphopoietin (TSLP). All these mediators are now well known for taking part in the remodeling of the airway’s activation and subsequent wheezing development [39]. Moreover, there are shreds of evidence about the fact that through Th2 cells activation, previous RV infection results are associated with the development of the so-called “indicators of atopy”: eosinophilia and allergen specific IgE [52]. RV bronchiolitis has been found statistically associated with eosinophilia, especially with a blood eosinophil count >400/mm^3^ [34,46], more frequently than non-RV bronchiolitis [19,28,30]. Moreover, as mentioned earlier, Amat and colleagues in 2018 found a correlation between co-infected RV + RSV bronchiolitis and the increased risk of sensitization to aeroallergens at three years at multivariate analysis [47]. The other peculiar RV-related immunological aspects are represented by the low IFN responses, especially IFN-γ. Low IFN responses in early life increase the likelihood of respiratory illnesses, including those associated with wheezing. Moreover, studies on airway epithelial cells cultured from patients with asthma have observed a diminished production of IFN-β, IFN-γ, and IFN-λ, which facilitates RV replication during infection. Reduced IFN-γ responses in infancy are also observed in “atopic children”, and this fact may contribute to explain why atopy is a risk factor for virus-induced wheezing [52]. Other studies have also identified differences in IFN-λ 1–3 levels in infants with RV or RSV bronchiolitis that may explain different clinical courses [28].

However, exposure to RV does not lead to asthma in all children, suggesting that personal risk factors (genetic, allergy, and antiviral immunity) and environmental exposure (farm, urban, microbes, and nutrition) also play a role. It remains to be elucidated whether RV bronchiolitis contributes to asthma development or is a marker of asthma susceptibility. In this sense, RV may be a revealing factor for those with early airway inflammation (i.e., epithelial barrier dysfunction, Th2 polarized inflammation), low IFN responses (i.e., impaired viral defense), and/or genetic variations (i.e., virus-specific risk genes, single nucleotide polymorphisms), acting as a clinically useful risk marker of asthma [53].

## 4. Conclusions

Several studies have investigated the link between RV bronchiolitis and short- and long-term outcomes, such as the future risk of subsequent asthma.

Regarding the studies focused on the course of bronchiolitis, many of them have supported that RV is associated with milder disease severity in comparison with other viral pathogens, especially RSV. On the contrary, in the majority of cases, the comparison between sole RV bronchiolitis and multiple viral co-infections does not suggest any significant difference concerning the severity of illness. However, not all research studies are consistent with these results. Moreover, the great heterogeneity of the investigations—from study design to analyzed outcome (CSI, ventilatory support, PICU admission, and LOS)—implies a certain obstacle to any interpretation of the literature data. Comprehensively, the available data are insufficient to draw conclusions in this regard. Thus, to date, the detection of the responsible virus pathogen does not seem to significantly impact the prognosis of bronchiolitis. Future studies should focus on RV types and viral load, which may play a role in the clinical course of bronchiolitis.

On the contrary, regarding long-term clinical outcomes, many studies accordingly have reported a strong association between RV bronchiolitis and the development of preschool wheezing and asthma. If confirmed in future trials, these findings may challenge the notion that the viral etiology of bronchiolitis does not affect clinical outcomes and support further research to guide therapeutic strategies to prevent the development of asthma.

## Figures and Tables

**Table 1:** Principal studies evaluating the correlation between rhinovirus and severity of illness in children with bronchiolitis.

Study, Year, Country	Study Design, Study Population	Period of Study Enrollment	Age Group	Sample Size Number of Cases		Outcome—Measure of Interest	Results (*p*-Value)
Papadopoulos NG et al., 2002, Greece [15]	Prospective single-center, hospitalized children with a clinical diagnosis of bronchiolitis	Oct 1999–Sep 2000	<18 month	118 children, 87 virus-positive cases: Viral types - RSV 63/87 (72.4%) - RV 25/87 (28.7%) - Others 18/87 (20.7%) Co-infections - Coinfect. 16/87 (19.5%) - RV + RSV 10/16 (62.5%)		15-points CSI based on heart rate, respiratory rate, cyanosis, difficult in feeding, and oxygen saturation	RV associated with a higher CSI on admission at the univariate analysis (OR 5.4; 95% CI 1.7–17.2; *p* = 0.004) and at the multivariate logistic regression analysis (OR 4.9; 95% CI, 1.2–18.7; *p* = 0.022)
Richard N, 2008, France [16]	Retrospective single-center, hospitalized children with a clinical diagnosis of bronchiolitis	Sep 2003–Apr 2005 (2 winter seasons)	<1 year	180 infants, 173 virus-positive cases: Viral types - RSV 130/173 (75.1%) - RV 39/173 (22.5%) - Others 36/173 (20.8%) Co-infections - Coinfect. 44/173 (25.4%) - RV + RSV 13/44 (29.5%)		Admission to PICU Hospital LOS	Viral co-infections associated with an increased risk of admission to PICU at the univariate analysis (OR 2.9; 95% CI 1.4–6.4) and at the multivariate logistic regression analysis (OR 2.7; 95% CI, 1.2–6.2; *p* = 0.02) LOS tended to be prolonged in co-infection, but no statistically significant difference
Marguet C et al., 2009, France [17]	Prospective multicenter, hospitalized children with a clinical diagnosis of bronchiolitis	Nov 2002–Mar 2004 (3 winter seasons)	<1 year	209 infants, 198 virus-positive cases: Viral types - RSV 134/198 (67.7%) - RV 56/198 (28.3%) - Others 32/198 (16.2%) Co-infections - Coinfect. 49/198 (24.7%) - RV + RSV 30/49 (61.2%)		Duration of oxygen requirement Hospital LOS > 5 d	At the multivariate logistic regression, RV infection decreased the risk to LOS > 5 d compared to other viral infections, especially RSV-infection (OR 0.11, 95% CI 0.03-0.37) At the multivariate logistic regression, RV infection decreased the oxygen requirement compared to RSV-infections (OR 0.29, 95% CI 0.09–0.90)
Calvo C et al., 2010, Spain [18]	Prospective single-center, hospitalized children with a clinical diagnosis of bronchiolitis	Sep 2005–Jul 2008	<2 years	318 children, 275 virus-positive cases: Viral types - RSV 195/275 (61.3%) - RV 64/275 (17.4%) - Others 109/275 (39.6%) Co-infections - Coinfect. 79/275 (28.7%) N.B. frequency of RV+RSV infection not specified		Hospital LOS	No significant differences between RV and RSV single infections, nor between co-infections and single infections
Midulla F et al., 2010, Italy [19]	Prospective single-center, hospitalized children with a clinical diagnosis of bronchiolitis	Sep 2004–May 2007 (3 winter seasons)	<1 year	182 infants, 104 virus-positive cases: Viral types - RSV 75/104 (72.1%) - RV 16/104 (15.4%) - Others 26/104 (25%) Co-infections - Coinfect. 16/104 (15.4%) - RV + RSV 0		8-points CSI based on respiratory rate, nasal flaring or chest retractions, difficult in feeding, arterial oxygen saturation in room air Hospital LOS	RV infection bronchiolitis had a lower CSI at admission than RSV infection (3.00 ± 2.0 vs. 4.3 ± 2.4, *p* = 0.05) RV infection had a lower LOS than RSV-infection (4.0 ± 1.6 d vs. 5.3 ± 2.4 d, *p* = 0.05)
Miller EK et al., 2011, USA [20]	Prospective single-center, hospitalized children with a clinical diagnosis of bronchiolitis	Fall 2004–Spring 2008 (4 winter seasons)	<1 year	455 infants: Viral types - RV 81/455 (17.8%) - Others 374/455 (82.2%) Co-infections - RV + other 40/455 (9%)		12-points CSI based on respiratory rate, nasal flaring or chest retractions, wheezing, oxygen saturation in room air Oxygen requirement Hospital LOS	RV infection associated with a lower median CSI than infants with other viruses (2.0, (IQR, 1.0–5.5) vs. 5.0 (IQR, 2.0–8.0), *p* < 0.001) RV infection associated with less requirement of supplemental oxygen than infants with other viral causes (18% vs. 51%, *p* < 0.001) No significant differences in LOS
Brand HK et al., 2011, the Netherlands [21]	Prospective single-center, hospitalized children with a clinical diagnosis of bronchiolitis	Nov 2006–Apr 2009 (3 winter seasons)	<2 years	142 children, 138 virus-positive cases: Viral types - RSV 104/138 (75.3%) - RV 43/138 (31.2%) - Others 64/138 (46.4%) Co-infections - Coinfect. 58/138 (42%) - RV + RSV 24/58 (41.4%)		Disease severity 3 groups: mild group (no hypoxia or feeding problems), moderate group (oxygen requirement, feeding problems), severe group (MV requirement)	Co-infections more frequently found in mild and moderate disease than in severe disease (56 and 44% respectively vs. 19%, *p* = 0.003)
Mansbach JM et al., 2012, USA [22]	Prospective multicenter cohort study (MARC), hospitalized children with a clinical diagnosis of bronchiolitis	Nov 2007–Mar 2010 (3 winter seasons)	<2 years	2207 children, 2068 virus-positive cases: Viral types - single RSV 1075/2068 (52%) - single RV 167/2068 (8.1%) N.B. frequency of other viral infection not specified Co-infections - Coinfect. 658/2068 (31.8%) - RV + RSV 287/2068 (13.9%) - RV + non-RSV 110/2068 (5%) -RSV + non-RV 227/2068 (11%)		Hospital LOS (cut-off ≥3 d) PICU admission CPAP/intubation requirement	RV only infection or RV + non-RSV infection associated with shorter LOS than RV + RSV co-infection (5% and 4% LOS ≥ 3 d vs. 18% LOS ≥ 3 d, *p* < 0.001) No significant differences in terms of PICU admission and CPAP/intubation requirement between viral groups
Ricart S et al., 2012, Spain [23]	Prospective single-center, hospitalized children with a clinical diagnosis of bronchiolitis	Oct 2007–Oct 2008	<1 year	418 infants, 410 virus-positive cases: Viral types - RSV 287/410 (70%) - RV 125/410 (30.5%) - Others 254/410 (62%) Co-infections - Coinfect. 189/410 (46.1%) - RV + RSV 56/189 (24.3%)		16-points CSI based on respiratory and heart rate, air entrance, retractions, wheezing, oxygen saturation in room air	RV infection associated with a severe CSI (≥11) than the other viral types at the univariate analysis (39% CSI ≥ 11 vs. 27.7% CSI < 11, *p* = 0.041), but no significant difference at the multivariate logistic regression analysis
Miller EK et al., 2013, USA [24]	Prospective single-center, children with a clinical diagnosis of bronchiolitis	Sep 2004–May 2008	<1 year	455 infants, 412 virus-positive cases Viral types - RSV 268/412 (65%) - RV 41/412 (10%) - Others 72/412 (17.5%) Co-infections - RV + RSV 30/412 (7.3%) N.B. frequency of global co-infections not specified		12-points CSI based on respiratory rate, nasal flaring or chest retractions, wheezing, oxygen saturation in room air	RV infection associated with lower CSI than RSV-infection (median 6.5 (IQR 3.9–8) vs. median 7 (IQR 5–9), *p* = 0.013)
Chen YW et al., 2014, Taiwan [25]	Prospective single-center, hospitalized children with a clinical diagnosis of bronchiolitis	Jan 2009–Mar 2011	<2 years	113 children, 86 virus-positive cases: Viral types - RSV 49/86 (57%) - RV 14/86 (16.3%) - Others 56/86 (65.1%) Co-infections - Coinfect. 28/86 (32.6%) N.B. frequency of RV+RSV infection not specified		Hospital LOS	No significant differences among RV infections and other single and mixed infections
Jartti T et al., 2014, Finland and USA [26]	Prospective multicenter cohort study (MARC), hospitalized children with a clinical diagnosis of bronchiolitis	Nov 2008–Mar 2010 (2 winter seasons)	<2 years	408 children, 350 virus-positive cases: Viral types - RSV 144/350 (41.4%) - RV 92/350 (26.3%) - Others 113/350 (32.3%) Co-infections - Coinfect. 63/350 (18%) - RSV + RV 10/63 (15.9%)		Hospital LOS (2 groups: <3 d and ≥3d)	Overall RV infection associated with shorter hospital LOS compared to RSV-infection (adjusted OR 0.45, 95% CI 0.22–0.92, *p* = 0.03)
Mehta R et al., 2014, USA [27]	Prospective cross-sectional single-center, hospitalized children with a clinical diagnosis of bronchiolitis	Oct 2010–Apr 2011	<2 years	131 children, 120 virus-positive cases: Viral types - RSV 84/120 (70%) - RV 34/120 (28.3%) - Others 37/120 (30.8%) Co-infections - Coinfect. 39/120 (32.5%) - RV + RSV 18/39 (46.2%)		Admission to PICU	No significant differences between types of viral infection about admission to PICU
Selvaggi C et al., 2014, Italy [28]	Prospective single-center, hospitalized children with a clinical diagnosis of bronchiolitis	2008–2011 (3 epidemic seasons)	<1 year	250 infants, 118 single RV or RSV-infection Viral types - RSV 78/118 (66.1%) - RV 40/118 (33.9%) - RV-A 18/40 (45%) - RV-C 22/40 (55%)		Hospital LOS	No significant differences in term of LOS between RV infection, RV types, and RSV bronchiolitis
Jartti et al., 2015, Finland and USA [29]	Prospective multicenter cohort study (MARC), hospitalized children with a clinical diagnosis of bronchiolitis with a focus on RV genomic viral load	2007–2010 (4 winter seasons, USA) 2008–2010 (3 winter seasons, Finland)	<2 years	2615 children, 694 with RV detection Viral types - RV 694/2615 (26.5%) - RV only 259/694 (37.3%) Co-infections - RV + RSV 297/694 (43%) - RV + non RSV 138/694 (20%)		Hospital LOS (cut-off ≥3 d) PICU admission CPAP/intubation requirement N.B. analyzed outcomes after classification of RV genomic load into 3 groups (low, intermediate, high)	No significant association between RV genomic load, nor co-infection, and LOS ≥ 3 days or risk of PICU admission or CPAP/intubation requirement in unadjusted and multivariable models
Cangiano G et al., 2016, Italy [30]	Prospective single-center, hospitalized children with a clinical diagnosis of bronchiolitis	Oct 2004–Mar 2014 (10 winter seasons)	<1 year	723 infants, 351 virus-positive cases: Viral types - RSV 234/351 (66.7%) - RV 44/351 (12.5%) - Others 34/351 (9.7%) Co-infections - Coinfect. 39/351 (11.1%) - RV + RSV 14/39 (35.9%)		8-points CSI based on respiratory rate, chest retractions, difficult in feeding, arterial oxygen saturation on room air Hospital LOS	No significant differences in terms of clinical severity score and LOS between different viruses’ group, including co-infected infants
Skjerven HO et al., 2016, Norway [31]	Prospective multicenter, hospitalized children with a clinical diagnosis of moderate-severe bronchiolitis based on a 10-points CSI	Jan 2010–May 2011 (2 winter seasons)	<1 year	363 infants, 330 virus-positive cases: Viral types - RSV 300/330 (90.9%) - RV 122/330 (37%) - RV A/B 35/122 (28.7%) - RV C 87/122 (71.3%) - Others 314/330 (95.2%) Co-infections - Coinfect 223/330 (67.6%) - RV + RSV 105/233 (45%)		Hospital LOS Level of supportive care (3 groups: no supportive care, use of oxygen and/or nasogastric tube feeding, use of ventilatory support)	No significant differences in terms of LOS nor level of supportive care between RV infection, RV types, other single virus-infections, RV + RSV infections, other co-infections
Paul SP et al., 2017, UK [32]	Prospective single-center, hospitalized children with a clinical diagnosis of bronchiolitis	Apr 2012–Dec 2015	<2 years	319 children, 227 single RV or RSV-infection Viral types - RSV 162/227 (71.4%) - RV 65/227 (28.6%)		Hospital LOS	RV infection associated with a longer hospital LOS than RSV-infection (4.6 d (range 0–21 d) vs. 3.2 d (range 0–13 d), *p* = 0.032)
Garcìa-Garcìa ML et al., 2017, Spain [33]	Prospective cross-sectional single-center, hospitalized children with a clinical diagnosis of bronchiolitis	Oct 2013–Apr 2016	<2 years	213 hospitalized children vs. 45 healthy controls, 186 virus-positive cases: Viral types - RSV 149/186 (80.1%) - RV 42/186 (22.6%) - Others 42/186 (22.6%) N.B. frequency of co-infections nor RV + RSV infection not specified		Hospital LOS	No significant differences among RV bronchiolitis and RSV-bronchiolitis
Petrarca L et al., 2018, Italy [34]	Retrospective single-center, hospitalized children with a clinical diagnosis of bronchiolitis	Oct 2004–May 2016 (12 winter seasons)	<1 year	486 virus-positive infants: Viral types - RSV 365/486 (75.1%) - RV 89/486 (18.3%) Co-infections - Coinfect. 54/486 (11.1%) - RV + RSV 20/54 (37%)		8-points CSI based on respiratory rate, nasal flaring or chest retractions, difficult in feeding, arterial oxygen saturation in room air PICU admission Hospital LOS Oxygen requirement	No significant differences about clinical severity score, PICU admission, LOS, oxygen requirement between RSV-infection, RV infection, RV + RSV infection
Praznik A et al., 2018, Slovenia [35]	Retrospective single-center, children with a clinical diagnosis of bronchiolitis with focus on the need for hospitalization (stratification in 3 groups of patients: outpatients with no hospitalization, standard hospitalization, admission to PICU)	May 2014–Apr 2015	<2 years	761 children, 473 virus-positive cases: Viral types - RSV 272/473 (57.5%) - RV 121/473 (25.6%) - Others 87/473 (18.4%) Co-infections - Coinfect. 151/473 (31.9%) N.B. frequency of RV+RSV infection not specified		Disease severity classified according to the site management (3 groups: outpatients, standard hospital, PICU)	No significant differences between types of viral infection about patient’s site management
Bergroth E et al., 2019, Finland [36]	Prospective multicenter cohort study (MARC-30), hospitalized children with a clinical diagnosis of bronchiolitis with follow-up 4 years after discharge	Nov 2008–Mar 2010	<2 years	349 children, 302 virus-positive cases: Viral types and RV types - RSV 145/302 (48%) - RV 101/302 (33.4%) - RV-A 24/101 (23.8%) - RV-B 3/101 (3%) - RV-C 73/101 (72.3%) - Others 51/302 (16.9%) Co-infections - RSV + RV 8/302 (2.6%) N.B. frequency of co-infections not specified		Hospital LOS (cut-off ≥ 3 d) Admission to PICU	RV-A and RV-C infection associated with lower LOS than RSV-infection (25% and 19% vs. 41%, *p* = 0.003) RV infection associated with lower admission to PICU than RSV-infection (0 vs. 9%, *p* = 0.02)
Hasegawa K et al., 2019, Finland and USA [37]	Prospective multicenter cohort study (MARC-30, Finland and MARC-35 USA), hospitalized children with a clinical diagnosis of bronchiolitis with a focus on RVspecies	2008–2010 (2 winter seasons, Finland) Nov 2011–Apr 2014 (3 winter seasons, USA)	<1 year	MARC-30 Finland 408 infants, 109 RVinfection RV types RV-A (23%) RV-B (3%) RV-C (74%) Co-infections RV + RSV (7%)	MARC-35 USA 1016 infants, 197 RVinfection RV types RV-A (47%) RV-B (6%) RV-C (47%) Co-infections RV + RSV (56%)	Hospital LOS PICU admission CPAP/intubation requirement	No significant differences in terms of LOS, PICU admission, and CPAP/intubation requirement between the 3 different RV types

CI = confidence interval; CPAP = continuous positive airway pressure; CSI = clinical score index; d = day; LOS = length of stay; MARC = Multicenter Airway Research Collaboration; mo = month; MV = mechanical ventilation; OR = odds ratio; PICU = Pediatric Intensive Care Unit; RSV = respiratory syncytial virus; RV = rhinovirus; y = year.

**Table 2:** Principal studies evaluating the correlation between rhinovirus bronchiolitis and preschool wheezing or asthma.

Study, Year, Country	Study Design, Study Population	Period of Study Enrollment	Age Group	Sample Size Number of Cases	Outcome—Measure of Interest	Results (*p*-Value)
Koponen P et al., 2011, Finland [45]	Prospective single-center, hospitalized children with a clinical diagnosis of bronchiolitis with a follow-up at 5-6 years of age	Dec 2001–May 2002 and Oct 2002–May 2004	<6 months	166 infants: Viral types - RSV 117/166 (70.5%) - RV 21/166 (12.7%) - Others 28/166 (16.9%)	Current asthma, defined as a continuous maintenance medication for asthma, doctor-diagnosed wheezing or prolonged (4 weeks) cough apart from an infection, during the preceding year and BHR in ECT	Previous non-RSV bronchiolitis associated with more frequent current asthma in preschool-age than RSV bronchiolitis (24% vs. 8.2%, *p* = 0.01), but no single virus (nor RV) was predominant in the “former non-RSV bronchiolitis” group with current asthma At the adjusted multivariate analysis, non-RSV bronchiolitis resulted as an independent risk factor for preschool asthma (aOR 3.74, 95% CI 1.28–10.99)
Midulla F et al., 2014, Italy [46]	Prospective single-center, hospitalized children with a clinical diagnosis of bronchiolitis with yearly follow-up for 3 years since discharge	Oct 2004–May 2008	<1 year	230 infants, 134 virus-positive cases: Viral types - RSV 99/134 (73.9%) - RV 20/134 (14.9%) - Others 29/134 (21.6%)	Recurrence of wheezing (3 groups: no wheezing, occasional wheezing (≥2 episodes of wheezing in 3 years), recurrent wheezing (≥2 episodes of wheezing in a year for3 years))	RV bronchiolitis associated with recurrent wheezing than other viral groups at multivariate analysis (OR 3.2, 95% CI 1.1-9.6, *p* = 0.03)
Amat et al., 2018, France [47]	Prospective single-center, hospitalized children with a clinical diagnosis of bronchiolitis with follow-up for 3 years after discharge	Oct 2011–May 2012	<1 year	154 infants: Viral types - RSV 117/154 (76%) - RV 44/154 (28.6%) - Others 108/154 (70.1%) Co-infections - Coinfect. 54/154 (35.1%) N.B. frequency of RV + RSV infection not specified	Diagnosis of recurrent wheezing Sensitization (IgE rate) to aeroallergens	No significant differences in the frequency of recurrent wheezing among single and mixed viral groups, but initial RV-RSV co-infected bronchiolitis associated with the risk of sensitization to aeroallergens at 3 years at multivariate analysis (OR 3.88, 95% CI 1.24–12.1, *p* = 0.02)
Bergroth E et al., 2019, Finland [36]	Prospective multicenter cohort study (MARC-30), hospitalized children with a clinical diagnosis of bronchiolitis with follow-up 4 years after discharge	Nov 2008–Mar 2010	<2 years	349 children, 302 virus-positive cases: Viral types - RSV 145/302 (48%) - RV 101/302 (33.4%) - Others 51/302 (16.9%) Co-infections - RSV + RV 8/302 (2.6%) N.B. frequency of global co-infections not specified	Use of asthma control medication 4 years after bronchiolitis	RV infection associated with higher use of asthma control medication in the last year than RSV infection (47% vs. 15%, aOR 3.67, 95% CI 1.88–7.19, *p* < 0.001) At the multivariable analysis, RV-C was specifically associated with a higher risk for the use of asthma control medication 4 years after severe bronchiolitis (aOR 3.72, 95% CI 1.80–7.66, *p* < 0.001)
Hunderi JOG et al., 2020, Norway [48]	Prospective multicenter, hospitalized children with a clinical diagnosis of moderate-severe bronchiolitis (based on a 10-pointed CSI) with follow-up 2 years after discharge	2010–2011	<2 years	294 children, 129 virus-positive cases Viral types - RSV 107/129 (82.9%) - RV 45/129 (34.9%) - RV-A/B 12/45 (26.7%) - RV-C 33/45 (73.3%) - high RV genomic load 8/45 (17.8%) Co-infections - Coinfect. 170/266 (63.9%) N.B. frequency of RV + RSV infection not specified	Recurrent wheeze at 2 years (defined as ≥3 episodes, including the bronchiolitis at the enrollment)	Recurrent wheeze by 2 years of age was neither significantly associated with RV or RSV nor with rates of high viral load during acute infant bronchiolitis
Mansbach et al., 2020, USA [49]	Prospective multicenter cohort study (MARC-35), hospitalized children with a clinical diagnosis of bronchiolitis with follow-up 2 years after discharge with a focus on delayed viral clearance (detection of the same virus in nasal swab 3 weeks after the discharge) and sequential infection (detection of a new RSV type or RV genotype)	2011–2014 (winter seasons)	<1 year	673 infants: Viral typed - RSV 107/673 (15.9%) - RV 122/673 (18.1%) Co-infections - Coinfect. 170/266 (63.9%) N.B. frequency of RV + RSV infection not directly specified	Recurrent wheezing by age 3 years (defined as having ≥2 corticosteroid-requiring exacerbations in 6 months or ≥4 wheezing episodes in 1 year that last ≥1 day and affect sleep)	No significant association between RSV DC, RSV SI, RV DC, RV SI, and a higher risk of recurrent wheezing Among infants with RV at hospitalization, those with a new RV SI had a higher risk of recurrent wheezing compared to children without a new RV SI (HR 2.49, 95% CI 1.22–5.06, *p* = 0.01)

aOR = adjusted odds ratio; BHR = bronchial hyper responsiveness; CI = confidence interval; CSI = clinical score index; DC = delayed clearance; ECT = exercise challenge test; HR = hazard ratio; IgE = immunoglobulin E; MARC = Multicenter Airway Research Collaboration; mo = month; RSV = respiratory syncytial virus; RV = rhinovirus; SI = sequential infection; y = year.
